# Radiogenomic approach combining CT-based radiomics and liquid biopsy improves prognostic stratification in patients with advanced NSCLC

**DOI:** 10.1016/j.jlb.2026.100470

**Published:** 2026-04-26

**Authors:** Alberto Ranghiero, Lisa Rinaldi, Sofia Netti, Aurora Gaeta, Ilaria Attili, Gianluca Spitaleri, Gianluigi Funicelli, Serena Mora, Cristiana Fodor, Daniela Anna Origgi, Filippo De Marinis, Nicola Fusco, Stefania Rizzo, Massimo Barberis, Sara Raimondi, Francesca Botta, Sara Gandini, Elena Guerini-Rocco

**Affiliations:** a Division of Pathology, IEO European Institute of Oncology IRCCS, Milan, Italy; b Division of Medical Physics, IEO European Institute of Oncology IRCCS, Milan, Italy; c Department of Experimental Oncology, IEO European Institute of Oncology IRCCS, Milan, Italy; d Philogen S.p.A, Italy; e Department of Statistics and Quantitative Methods, University of Milano-Bicocca, Milan, Italy; f Division of Thoracic Oncology, IEO European Institute of Oncology IRCCS, Milan, Italy; g Division of Radiology, IEO European Institute of Oncology IRCCS, Milan, Italy; h Data Management Unit, IEO European Institute of Oncology IRCCS, Milan, Italy; i Department of Oncology and Hemato-Oncology, University of Milan, Milan, Italy; j Clinic of Radiology EOC, Institute of Integrated Diagnostics of Southern Switzerland (IDISI), Lugano, Switzerland; k Faculty of Biomedical Sciences, Università Della Svizzera Italiana (USI), Lugano, Switzerland; l Medical Physics Dept. ASST-Settelaghi, Varese, Italy

**Keywords:** Non-small cell lung cancer (NSCLC), Radiomics, Liquid biopsy, ctDNA, Radiogenomics, Prognostic stratification, Targeted therapy

## Abstract

**Background:**

Radiomics and liquid biopsy represent minimally invasive approaches to assess disease characteristics in solid tumors. We integrated computed tomography (CT) radiomics and circulating tumor DNA (ctDNA) analysis to enhance prognostic stratification and longitudinal monitoring in patients with advanced non-small cell lung cancer (NSCLC).

**Methods:**

This study prospectively enrolled 91 patients with advanced NSCLC. Baseline molecular profiling was performed on both tumor tissue and plasma ctDNA using targeted next-generation sequencing. Radiomic features were extracted from baseline CT lung lesions using PyRadiomics, and radiomic scores (RS) were developed using LASSO-regularized Cox models. A subgroup of 21 patients with actionable molecular alterations underwent longitudinal CT scans and liquid biopsies during targeted therapy. Clinical, radiomic, and molecular associations with overall survival (OS) and disease-free survival (DFS) were evaluated using log-rank tests and included in multivariable models.

**Results:**

Overall concordance between tissue and ctDNA (n = 67 patients) was 85%. In the combined clinical-radiomic-genetic model (C-index: 0.73), the RS (p < 0.001) and the presence of actionable alterations (p = 0.041) were independent OS predictors. For DFS, the integrated model achieved a cross-validated C-index of 0.77, outperforming the clinical-only model (0.59). In patients with EGFR-mutant NSCLC, detectable baseline ctDNA was significantly associated with a higher risk of disease progression (p = 0.018). In this subgroup, the combined clinical-radiogenomic model achieved a cross-validated C-index of 0.80 for DFS. Longitudinal analysis showed that 17 of 21 patients achieved molecular clearance of ctDNA at the first follow-up, correlating with treatment response.

**Conclusions:**

Integrating radiomics with liquid biopsy provides a more robust prognostic assessment of advanced NSCLC than clinical or molecular data alone. This multi-modal approach may offer a minimally invasive strategy for personalized risk stratification and monitoring of treatment response in patients with NSCLC.

Clinicaltrials.gov identifier: NCT06331975.

## Highlights

• Integrated radiogenomic models outperform clinical-only models in predicting OS and DFS in advanced NSCLC.
• High concordance (85%) was observed between tissue biopsy and ctDNA for molecular profiling at diagnosis.
• Baseline ctDNA status is a significant independent prognostic factor, particularly in EGFR-mutant patients.
• Radiomic scores (RS) derived from standard CT scans provide independent prognostic value for survival outcomes.
• Longitudinal ctDNA monitoring effectively tracks response and disease progression during targeted therapy.

## 1 Introduction

Lung cancer remains the leading cause of cancer-related mortality globally, with non-small cell lung cancer (NSCLC) comprising approximately 85% of cases [1,2]. Molecular profiling has revolutionized NSCLC management by enabling tailored treatments, with a demonstrated improvement in patient survival [3]. However, prognosis and response to therapy remain heterogeneous even within molecularly defined subgroups. An in-depth characterization of NSCLC heterogeneity is therefore crucial for refining patient prognostic and predictive stratification.

Traditionally, molecular profiling for advanced NSCLC relies on small biopsy or cytology specimens from a single tumor site. These samples, while informative, represent a spatial and temporally limited snapshot of the tumor, and they may not fully capture tumor heterogeneity or provide real-time insights into disease evolution [4]. These limitations underscore the need for minimally invasive alternatives that offer a more comprehensive and dynamic assessment of tumor characteristics.

Liquid biopsy, specifically analyzing circulating tumor DNA (ctDNA) in plasma, is now a valid non-invasive option for molecular profiling in advanced NSCLC, allowing for the assessment of tumor mutational status and disease monitoring over time [5]. Additionally, radiomics, which extracts quantitative features from standard radiological images, is being investigated as a complementary non-invasive tool to enhance NSCLC diagnosis, prognostication, and prediction of treatment response [6]. Despite their promise, both liquid biopsy and radiomics have inherent limitations. ctDNA analysis can exhibit lower sensitivity for detecting certain alterations, particularly in cases of low tumor burden [7]. Similarly, radiomic models face challenges regarding reproducibility and robust validation [8,9].

The integration of radiomics and genomic information, often referred to as “radiogenomics,” holds potential to overcome these limitations. By combining quantitative image features with genetic information derived from ctDNA, radiogenomics may provide a more comprehensive, minimally invasive assessment of disease behavior, enhancing patient prognostication and selection for targeted therapies. Our group has recently shown that contrast-enhanced computed tomography (CT)-based radiomics might improve the prognostic stratification of patients with NSCLC and provided some evidence of an association between NSCLC mutational status and CT-based radiomics [10]. In this study, we performed molecular profiling on ctDNA and CT-based radiomics analysis in a prospective cohort of patients with advanced NSCLC, aiming (i) to investigate the prognostic role of baseline liquid biopsy status in combination with radiomics features and (ii) to explore the potential role of liquid biopsy and radiomics in monitoring during targeted therapy.

## 2 Material and methods

### 2.1 Study design and study population

This prospective, single-center study enrolled 128 patients with advanced non-small cell lung cancer (NSCLC) who underwent diagnostic procedures at the European Institute of Oncology from 2019 to 2023. Patients were eligible if they had histologically or cytologically confirmed advanced non-squamous NSCLC. Exclusion criteria included insufficient tumor specimens for molecular analysis or unavailable CT imaging at the time of diagnosis. The study was approved by the Institutional Review Board, and all patients provided written informed consent before participation. Clinical data, including age, sex, smoking history, disease stage at enrollment, treatments received, disease progression status, survival status (updated to August 2023), and dates of follow-up assessments, were collected.

### 2.2 Baseline tumor tissue molecular profiling

Genomic analysis was performed on tumor tissue obtained from diagnostic biopsy or cytology samples. DNA and RNA were extracted using the Promega Maxwell RSC DNA and RNA FFPE kit (Promega, Madison, WI, USA) on the Promega Maxwell automated extractor. Molecular profiling was conducted using the NGS Oncomine Comprehensive Assay panel (Thermo Fisher Scientific, Waltham, MA, USA), as previously reported [11]. Briefly, 10 ng of genomic DNA and RNA were processed on the Ion Chef automated platform (Thermo Fisher Scientific, Waltham, MA, USA) for library preparation. The sequencing run was performed on Ion S5 System (Thermo Fisher Scientific, Waltham, MA, USA) and data were analyzed using the Ion Reporter Analysis Software v5.10. Only alterations with a Variant Allele Frequency (VAF) equal/superior to 5% and with adequate quality metrics (read depth >100; VAF x read depth >25; p-value = 0.00001 and Median of the Absolute values of all Pairwise Difference (MAPD) < 0.5) were considered for further analyses. For clinical stratification, we defined the presence of actionable alterations as the detection of druggable variants in key NSCLC oncogenes, including *EGFR*, *KRAS* G12C, *BRAF*V600, *ERBB2* mutations, *MET* exon 14 skipping and *ALK*, *ROS1*, *RET* rearrangements.

### 2.3 ctDNA analysis

Eight ml of plasma was collected from each patient at enrollment using dedicated collection tubes for cell-free DNA (cfDNA) isolation. cfDNA was extracted using the AVENIO cfDNA Isolation Kit (Roche Molecular Systems, Inc., Branchburg, New Jersey, USA; For Research Use Only) and sequenced with the AVENIO ctDNA Targeted Kit (Roche Molecular Systems, Inc., Branchburg, New Jersey, USA; For Research Use Only), following the manufacturer's instructions. Sequencing was performed on the NextSeq 500 platform (Illumina, San Diego, CA), and data were analyzed using the AVENIO Oncology Analysis Software bioinformatics pipeline. Concordance between molecular data from tumor tissue/cytology samples and liquid biopsy was assessed for each patient.

For patients harboring actionable molecular alterations and receiving targeted therapy, liquid biopsy sampling was further conducted every 3–6 months or at the time of disease progression to monitor genomic evolution during treatment. cfDNA was extracted from plasma using the AVENIO cfDNA Isolation Kit and sequenced with the AVENIO ctDNA Expanded Kit (Roche Molecular Systems, Inc., Branchburg, New Jersey, USA; For Research Use Only), following the manufacturer's instructions. Sequencing was performed on the NextSeq 500 platform, and data were processed using the AVENIO Oncology Analysis Software (Roche Diagnostics, Mannheim, Germany; For Research Use Only). For each case, the persistence, absence, and changes in allele frequency (variant allele fraction, VAF) of the actionable alteration identified at enrollment were evaluated.

### 2.4 Radiomic analysis

For each patient enrolled, a contrast-enhanced lung CT study was acquired at baseline and portal phase images were considered for radiomic analysis. An automatic lung lesion segmentation algorithm, developed using deep learning techniques, was adopted for lesion segmentation on CT images. Specifically, a nnU-Net model was trained on a combination of retrospective patient data and publicly available datasets, with manually delineated contours by physicians serving as the gold standard. The performance of the automatic segmentation model was assessed through geometric comparisons between manually and automatically segmented volumes. Additionally, the potential impact of segmentation discrepancies on radiomic model performance was evaluated. The results demonstrated a high degree of concordance between manual and automated volumes, with no significant differences in radiomic model outcomes. (Data not shown/[12]). The images and the lesion volume files were downloaded as DICOM (Digital Imaging and Communications in Medicine) format and then converted to NRRD (Nearly Raw Raster Data) format for radiomic feature extraction. Radiomic features were extracted with the PyRadiomics v2.2.0 package as previously reported [10]. Only features demonstrating stability and reproducibility were considered for further analyses [8]. For patients with actionable molecular alterations and receiving targeted therapies, longitudinal radiomic evolution during targeted therapy was assessed on serial CT scans, following the methodology described above for baseline imaging.

### 2.5 Statistical analysis

Concordance between molecular alterations detected in liquid biopsy (circulating tumor DNA, ctDNA) and matched tumor tissue or cytology samples at diagnosis was evaluated using descriptive statistics, Cohen's kappa coefficient, and McNemar's chi-squared test. The main endpoints were disease-free survival (DFS) and overall survival (OS). For each outcome, associations with clinical, radiomic, and molecular features were first assessed using log-rank tests and subsequently included in multivariable Cox proportional hazards regression models. Hazard ratios (HRs) and 95% confidence intervals (CIs) were reported. A radiomic score (RS) was developed for both OS and DFS and internally validated. Details on the RS development are reported in Supplementary Methods.

A subgroup analysis was performed in patients with *EGFR* mutations and complete follow-up data. Clinical and genetic models were developed using the same analytical pipeline, incorporating RS information from the overall cohort when applicable. If the RS derived from the overall cohort was not statistically significant in this subgroup, an exploratory feature-level analysis was conducted to identify radiomic predictors of prognosis.

Longitudinal radiomic evolution under targeted therapy was evaluated using serial CT scans and paired liquid biopsies. Baseline and follow-up RS values were compared, and their change (ΔRS) was evaluated using a Cox model for both DFS and OS, adjusted for the interval between scans. Additionally, delta radiomic analyses at the feature level were performed by computing changes in individual features (follow-up minus baseline), with the same pipeline described in Supplementary Methods.

## 3 Results

A total of 91 patients were included in the final analysis. The main clinicopathological characteristics of this analyzed cohort are summarized in Table 1.

**Table 1:** Clinicopathological characteristics of the study population.

Table 1	N = 91
Sex	
Female	45 (49%)
Male	46 (51%)

Age	67 (59, 72)
Smoke	
Current smoker	18 (20%)
Ex smoker	41 (47%)
Not a smoker	29 (33%)
Missing	3
Primitive cancer site	
Right lower lobe	13 (15%)
Left lower lobe	8 (9.0%)
Median lobe	2 (2.2%)
Right upper lobe	30 (34%)
Left upper lobe	17 (19%)
Mix	19 (21%)
Missing	2
Stage	
III	4 (4.4%)
IV	87 (96%)
Encephalic Metastases	
No	62 (68%)
Yes	29 (32%)

### 3.1 Concordance between cytology/tissue and liquid biopsy sample molecular profile at diagnosis

Molecular profile data for the concordance analysis between tissue and liquid biopsy (ctDNA) were available for 67 cases. Considering all types of actionable alterations, the overall concordance rate was 85% (Kappa (95%CI): 0.62 (0.42-0.83); McNemar's test p-value: 0.06).

When examining individual recurrent actionable gene alterations, *EGFR* mutation analysis showed an overall concordance of 91% (McNemar's test p-value: 0.10; Kappa (95%CI): 0.79 (0.63-0.95)). *KRAS* mutations (including *KRAS* G12C) demonstrated almost perfect agreement, with a concordance rate of 99% (Kappa (95%CI): 0.97 (0.90-1.00)). Discordances were observed for *ALK* and *ROS1* genes, where fusion genes were more frequently detected in tissue. However, these differences were not statistically significant.

### 3.2 Association of selected clinical, radiomic features and gene alterations at diagnosis with clinical outcomes

In the clinical analyses (Table 2), only smoking status was confirmed as an independent prognostic factor for overall survival (OS) (p = 0.007, cross-validated C-index: 0.62 (0.60–0.63)).

**Table 2:** Multivariable Cox proportional hazards analysis of overall survival across clinical, radiomic, and integrated radiogenomic models.

		n	Events n	HR	95% CI	p-value
Clinical model	*Smoke*					
	*Ex-No*	70	34	—	—	
	*Current*	18	13	2.46	1.29, 4.70	0.007
Clinical -Radiomic model	*Smoke*					
	*Ex-No*	69	34	—	—	
	*Current*	18	13	1.97	1.02, 3.78	0.042
	*RS*	87	47	5.33	2.92, 9.74	<0.001
Radiogenomic - Clinical model	*Smoke*					
	*Ex-No*	65	33	—	—	
	*Current*	16	12	1.63	0.81, 3.28	0.170
	*Actionable genes*					
	*Presence*	35	21	—	—	
	*Absence*	46	24	0.52	0.28, 0.97	0.041
	*RS*	81	45	5.16	2.79, 9.54	<0.001

HR hazard ratio and 95% Confidence Interval.

The multivariate LASSO-regularized Cox models identified a subset of radiomic features most strongly associated with OS, with corresponding coefficients used to calculate the RS (Supplementary Table 1). When radiomic data were integrated into a clinical-radiomic model, both smoking status (p = 0.042) and radiomic score (p < 0.001) remained significant, leading to an improved predictive performance for OS (Table 2; cross-validated C-index: 0.73 (0.71–0.75). In the combined clinical-radiomic-genetic model, the radiomic score (p < 0.001) and the presence or absence of actionable alterations (p = 0.041) remained independent predictors of OS. Notably, smoking status was no longer statistically significant in this comprehensive model (p = 0.170), while the model maintained robust predictive performance (Table 2; cross-validated C-index: 0.73 (0.72–0.74).

For disease-free survival (DFS; Table 3), the presence of brain metastases was significantly associated with a worse prognosis. The multivariate LASSO-regularized Cox models identified a subset of radiomic features most strongly associated with DFS, with corresponding coefficients used to calculate the RS (Supplementary Table 2). Both variables remained independent predictors of DFS across multivariable clinical, radiomic, and clinical-radiomic models. The cross-validated C-index values were 0.59 (0.57–0.60) for the clinical model, 0.73 (0.70–0.75) for the radiomic model, and 0.77 (0.75–0.80) for the clinical-radiomic model. These findings suggest that integrating radiomic data with clinical or genomic parameters enhances the ability to predict clinical outcomes.

**Table 3:** Multivariable Cox proportional hazards analysis of disease-free survival (DFS) across clinical – radiomic models.

		n	evenst n	HR^1^	95% CI^1^	p-value
Clinical Model	Brain metastases					
	No	34	21	—	—	
	Yes	17	12	2.37	1.09, 5.13	0.029
	RS	50	32	20.1	5.59, 72.3	<0.001
Clinical Radiomic model	Brain metastases					
	No	33	20	—	—	
	Yes	17	12	2.66	1.22, 5.81	0.014
	RS	50	32	21.7	6.10, 77.3	<0.001

HR hazard ratio and 95% Confidence Interval.

### 3.3 Prognostic relevance of liquid biopsy and radiomics in EGFR-mutant patients

A focused analysis on a homogeneous subgroup of 25 patients with EGFR-mutant NSCLC revealed a significant association between cfDNA status at baseline (presence or absence of *EGFR* mutation in baseline liquid biopsy), radiomic data, and clinical outcomes. Patients with detectable ctDNA (*EGFR* mutation-positive cfDNA) at baseline exhibited a significantly higher risk of disease progression compared to patients with undetectable ctDNA (p = 0.018). In the multivariable model for this subgroup, the presence of brain metastases (p = 0.006), a specific radiomic feature (Wavelet-HL glcm1 ClusterShade, p = 0.03), and cfDNA status (presence of *EGFR* mutation in liquid biopsy, p = 0.016) were identified as independent predictors of worse prognosis (Table 4). The combined clinical-radio-genomic model, incorporating these variables, demonstrated fair predictive accuracy for DFS, with a cross-validated C-index of 0.80 (0.74–0.85). This highlights the potential of integrating imaging and circulating biomarkers for risk stratification in NSCLC patients.

**Table 4:** Cox proportional hazards models (DFS) integrating clinical and radiogenomic features in EGFR-mutated NSCLC.

		N	Events n	hr^1^	95% ci^1^	P-value
Clinical	Brain metastases					
	No	13	9	—	—	
	Yes	7	6	8.74	1.86, 41.2	0.006
Clinical radiomic	Radiomic feature (Wavelet-hl glcm1 clustershade)	20	15	1.12	1.01, 1.24	0.033
	EGFR (cfDNA)					
	-	4	1	—	—	
	+	16	14	15.4	1.65, 143	0.016

HR hazard ratio and 95% Confidence Interval.

### 3.4 Longitudinal analysis of ctDNA and radiomics in patients receiving targeted therapy

Among 21 patients harboring actionable molecular alterations and receiving targeted therapies, longitudinal liquid biopsy monitoring was performed to assess dynamic molecular changes during treatment. This subgroup included 16 patients with *EGFR* mutations, three with *ALK* gene rearrangements, two with *RET* gene rearrangements, and one with a *ROS1* gene rearrangement. At enrollment, prior to treatment initiation, the actionable alteration identified in tissue biopsy was also detected in circulating free DNA (cfDNA) in 12 of 21 patients. However, at the first follow-up (T1) after initiating targeted therapy, the cfDNA-detected alteration was not detected in 17 of 21 patients, suggesting a positive treatment response at the molecular level. Longitudinal analysis revealed patient-specific variations in allele frequency (variant allele fraction, VAF) over time in cfDNA samples collected during therapy (Supplementary Fig. 1).

In the subgroup with *EGFR* mutations, the radiomic score (RS), calculated at baseline and at first follow-up, showed no significant association with clinical outcomes (data not shown). Among 16 patients with *EGFR*-mutated tumors who received front-line targeted treatment with osimertinib and underwent longitudinal monitoring through liquid biopsy, n = 5 had driver mutation undetectable and n = 11 had *EGFR* driver mutation detectable in baseline ctDNA. The best response was partial response (PR) in 14 out of 16 patients, whereas one patient experienced stable disease (SD) and one patient had progressive disease (PD) as the best response to osimertinib treatment. Of note, among the baseline *EGFR* undetectable patients, 3 out of 5 patients experienced long-term disease control (more than 3 years), with only one patient converting into *EGFR-positive* ctDNA at systemic disease progression. Conversely, only one patient with the *EGFR* ctDNA-positive had EGFR ctDNA-negative findings at PD. *EGFR* ctDNA negativization or VAF decrease was associated with PR in all cases, turning positive again at disease progression (Supplementary Fig. 1).

## 4 Discussion

This prospective, single-center study investigated the integration of radiomics and liquid biopsy to enhance prognostic stratification in a cohort of patients with advanced non-squamous non-small cell lung cancer (NSCLC). We found that multi-modal strategies significantly improve the prediction of patient outcomes. Additionally, our data underscores the established utility of liquid biopsy for both initial molecular diagnosis and longitudinal monitoring of disease.

At diagnosis, we observed an 85% overall concordance rate between tumor tissue and circulating tumor DNA (ctDNA) for molecular profiling, confirming the utility of liquid biopsy as a reliable, non-invasive alternative for initial testing in patients with advanced NSCLC [5,13]. High sensitivity was demonstrated for common driver mutations, with the strongest agreement seen for *EGFR* (91%) and *KRAS* (99%) mutations, including G12C. However, differences were noted, particularly for fusion gene variants, which were detected more often by tissue analysis. These findings highlight the complementary nature of tissue and liquid biopsy approaches [5]. The lower detection rate in ctDNA is likely due to both intrinsic biological and technical limitations. Technically, the short length of ctDNA fragments and the design of most DNA-only-based next-generation sequencing (NGS) panels present major challenges for detecting fusion genes in liquid biopsy [7]. Biologically, low ctDNA abundance from reduced tumor shedding or low tumor burden can decrease the informativeness of liquid biopsy testing [14]. In this case, as recommended, a negative result should prompt a reflex tissue testing to maximize the chance of finding druggable alterations [5,7].

Our study also provided evidence for the independent prognostic value of these biologically related discordances. Indeed, in the group of EGFR-mutant NSCLC by tissue analysis, patients with EGFR mutation-positive ctDNA at baseline had a significantly higher risk of disease progression compared to those with negative ctDNA status. This finding is consistent with previous data showing that detectable ctDNA reflects a higher tumor burden or a more aggressive disease, making it a valuable prognostic biomarker even before treatment initiation [5,15]. This evidence suggests that cfDNA status at baseline may be used to stratify patients with *EGFR* mutations into different risk groups, potentially guiding the intensity and frequency of subsequent monitoring or treatment strategies.

Our findings demonstrated that an integrated approach of radiogenomic - combining quantitative imaging features from radiomics with genetic information from liquid biopsy - may provide a more comprehensive assessment of disease characteristics and behavior. Recently, it has been shown that radiomics can enhance diagnostic accuracy, predict biomarker status, patient prognosis, and dynamically monitor treatment response [[16], [17], [18], [19]]. Our group recently demonstrated that CT-based radiomic signatures may predict *EGFR*, *KRAS* and *ALK* gene status in NSCLC [10]. Furthermore, we developed clinical-radiomic models that outperformed either clinical or radiomic models alone in the prediction of tumor molecular alterations and OS [10]. In this study, a combined clinical-radiomic model incorporating smoking status and a radiomic score (RS) achieved a cross-validated C-index of 0.73, superior to the clinical-only model (C-index: 0.62) for overall survival (OS). In the clinical-radiomic-genetic model, both the radiomic score and the presence or absence of actionable alterations were independent predictors of OS. Interestingly, smoking status was no longer statistically significant, probably due to specific genetic alterations that are often associated with non-smoking status [20]. Similarly, for disease-free survival (DFS), a combined clinical-radiomic model that included brain metastases and the radiomic score yielded a robust C-index of 0.77, significantly outperforming the clinical-only model's C-index of 0.59. These results show that radiomic features from standard CT scans contain valuable information about tumor biology that is not captured by conventional clinical or molecular markers.

This is further highlighted by our focused analysis on the EGFR-mutant subgroup. A combined clinical-radiogenomic model, including brain metastases, a specific radiomic feature (wavelet-HL glcm1 ClusterShade), and cfDNA status, showed a cross-validated C-index of 0.80 for DFS predictive accuracy. Similarly, Yousefi et al. (2021) developed a multivariable prognostic model for EGFR-mutated NSCLC that integrated radiomic features, circulating tumor DNA (ctDNA), and clinical variables, achieving a C-statistic of 0.77 for progression-free survival (PFS) [21]. Furthermore, emerging evidence suggests that specific *EGFR* mutation subtypes exhibit unique radiomic signatures [22,23]. EGFR-mutated NSCLCs represent a clinically and biologically heterogeneous disease [24]. Previous studies have shown differential expression patterns in genes related to cell proliferation and immune response between tumors harboring exon 19 deletion and exon 21 L858R mutation [25,26]. This mutation-specific influence on tumor growth and the microenvironment may manifest as distinct radiomic textures and features. While the limited sample size of the current study precluded an investigation of the radiomic impact and prognostic relevance of individual *EGFR* alterations, these data underscore the value of integrating diverse data types to create more accurate and specific predictive models for distinct patient cohorts, even within molecularly defined subgroups.

The study further investigated the role of liquid biopsy in dynamic monitoring throughout targeted therapy. Our longitudinal analysis of cfDNA in a cohort of 21 patients with actionable alterations revealed that 17 achieved molecular response, as evidenced by the clearance of ctDNA at the initial follow-up after treatment. This observation underscores the role of liquid biopsy as a minimally invasive tool for assessing early molecular response [27,28]. Moreover, given the feasibility of serial sampling, ctDNA is also emerging as a valuable method for real-time monitoring of treatment response [27,28]. Indeed, in our study, EGFR ctDNA negativization and VAF dynamics were associated with treatment response. The radiomic score did not show a significant association with clinical outcomes in this context. The lack of a significant association may be due to the limited number of patients and the specific radiomic features selected. Further research is warranted to explore more granular changes in individual radiomic features over time, as suggested by our delta radiomics analysis. A more detailed, feature-level analysis and a larger cohort could reveal more subtle but clinically meaningful associations between changes in tumor texture and treatment response. This study has several limitations, including its single-center design and relatively small sample size. This is particularly true for subgroups analyzed for specific mutations or longitudinal monitoring. These factors limit the generalizability of our findings and underscore the need for external validation in larger, multi-institutional cohorts to demonstrate their clinical value in different patient populations [9]. However, the study's prospective design mitigates selection bias and enables the collection of high-quality longitudinal data. Furthermore, a rigorous statistical approach, including internal cross-validation and corrections for multiple testing, was applied to ensure the robustness of our findings.

Despite the significant potential of integrated radiogenomic models, several barriers currently limit their widespread diffusion into routine clinical practice. A major hurdle is the high variability in image acquisition and segmentation, reconstruction algorithms, and feature extraction across different institutions, which can significantly impact the reproducibility of radiomic models [8]. Additionally, the implementation of these models requires standardized infrastructures and procedures for ctDNA analysis. Although liquid biopsy has already entered clinical practice as an alternative (or complementary) approach to traditional tumor tissue biopsy for molecular profiling of advanced-stage NSCLC, its availability and accessibility are still hampered by technical, socio-economic, and regulatory barriers [29].

## 5 Conclusion and future perspectives

In conclusion, our study demonstrates the significant potential of an integrated radiogenomic approach to enhance prognostic stratification in patients with advanced non-small cell lung cancer. We have shown that combining radiomic features from standard CT scans with genetic information from liquid biopsy provides a more comprehensive and robust prognostic model than either method alone. Furthermore, we validated the utility of ctDNA for both initial molecular profiling and for monitoring early molecular response to targeted therapies. While our findings underscore the value of this multi-modal strategy, future research should focus on larger, multi-institutional cohorts to confirm the generalizability of our integrated models and to further explore the potential of longitudinal radiomic and ctDNA changes as a means for real-time treatment monitoring and patient-specific risk stratification.

## Informed consent statement

Informed consent was obtained from all subjects involved in the study.

## Institutional Review Board statement

The study was conducted in accordance with the Declaration of Helsinki and approved by the Institutional Review Board of the European Institute of Oncology and by the local Ethics Committee (protocol code R784/18-IEO836; date of approval 29 May 2018).

## Author contributions

Conceptualization: MB, SR, FB, EG-R; Data curation: AR, SN, AG, LR, SM, CF; Formal analysis: AR, SN, AG, LR, IA; Funding acquisition: MB, SR; Investigation: AR, LR, IA, GS, GF, CR; Methodology: AR, SN, AG, LR, SRA; Supervision: SRA, FB, SG, EG-R; Roles/Writing - original draft: AR, SN, AG, IA, FB, EG-R; and Writing - review & editing: AR, SN, AG, LR, IA, GS, GF, SM, CF, CR, DAO, SR, MB, FDM, NF, SRA, FB, SG, EG-R.

## Declaration of generative AI in scientific writing

During the preparation of this manuscript, the Authors used the generative AI tool (Gemini Pro - Google) to assist with language refinement, formatting, and stylistic suggestions. Additionally, Grammarly (version 6.8.263), a natural language processing tool, was used for grammar checking. All content was reviewed and verified by the authors for accuracy and originality.

## FundingS

The study was funded by a research grant from the Italian Ministry of Health (Ricerca Finalizzata GR-2016-02362050), and partially supported by the Italian Ministry of Health with Ricerca Corrente and 5 X 1000 funds. LR, AR, MM, GLP, and GF were supported by a research grant from the Italian Ministry of Health (GR-2016-02362050). LR was also supported by the Fondazione IEO—Radiomic project. The analysis of ctDNA with AVENIO Targeted and Expanded Kits (Roche Molecular Systems, Inc., Branchburg, New Jersey, USA; For Research Use Only. Not for use in diagnostic procedures) and the cost of this publication was supported by Roche Molecular Systems, Inc., Pleasanton, CA, USA.

## Declaration of competing interest

SR has received travel grant from Bracco. MBA has received honoraria for consulting, advisory role, speakers’ bureau, travel, accommodation, expenses from MSD Oncology, Roche/Genetech, AstraZeneca, GSK, Amgen, Thermofisher Scientifics and Illumina. N.F. has received honoraria for consulting, advisory role, speaker bureau, travel, and/or research grants from Abbvie, Alira Health, AstraZeneca, Daiichi Sankyo, Epredia, Exact Sciences, Gilead, GSK, Leica Biosystems, Lilly, Menarini Group, Merck, MSD, Novartis, Pfizer, Roche, Sakura, Sysmex, ThermoFisher, Veracyte. E.G-R. has received advisory fees, honoraria, travel accommodations/expenses, grants, and/or non-financial support from AbbVie, AstraZeneca, Biocartis, Daiichi Sankyo, Exact Sciences, GE HealthCare, GSK, Illumina, Lilly, Novartis, MSD, Pfizer, Pillar Biosciences, Roche, Sophia Genetics, Stemline (Menarini), Thermo Fisher Scientific; All other authors declare no potential conflicts of interest.

Given his role as Editorial Board Member, Nicola Fusco had no involvement in the peer-review of this article and has no involvement in the peer review of this article and has no access to information regarding its peer review.

## Data Availability

Data will be made available upon reasonable request to the corresponding author.
