# Supplementary material for: Radiogenomic approach combining CT-based radiomics and liquid biopsy improves prognostic stratification in patients with advanced NSCLC
Source: J Liq Biopsy. 2026 Apr 26;12:100470. doi: 10.1016/j.jlb.2026.100470 (PMC13141799; doi:10.1016/j.jlb.2026.100470)

**SUPPLEMENTARY**

***Supplementary methods***

*Radiomic score*

Radiomic features with nearly zero variance and high correlation (Spearman ρ > 0.95) were excluded. To address multicollinearity and dimensionality, an iterative clustering process (ρ > 0.75) was employed to retain only the most outcome-associated feature from each cluster, based on the univariate log-rank test, and repeated until all the between-feature correlations (ρ) were ≤0.75. The multivariable LASSO-regularized Cox model was used to produce a coefficient for each feature associated with the outcome and then an overall score (RS). The integrated clinical–genetic–radiomic model was obtained by including the RS in the multivariate Cox framework. Model performance was evaluated using Harrell’s concordance index (C-index) with 4-fold internal cross-validation and 500 bootstrap resamples. The median C-index and interquartile range (IQR) were reported.

***Supplementary Tables***

Supplementary Table 1. Radiomic features retained in the multivariable LASSO-regularized Cox models with corresponding coefficients contributing to the radiomic score (RS) for overall survival (OS).

| Radiomic features | Coefficient |
| --- | --- |
| Original_glcm_7__Correlation | *1.4553* |
| Wavelet-LL_glcm1_MCC | *-1.4800* |
| Wavelet-LH_glcm4_MCC | *0.4105* |
| Wavelet-LH_glszm_graylevelvariance | *0.0419* |
| Wavelet-HL_glcm1_clustershade | *0.0142* |
| Wavelet-LH_glcm4_clusterprominence | *0.0009* |
| Log-sigma-2-5-mm-3D_glcm1_clustershade | *0.0002* |

Supplementary Table 2, Radiomic features retained in the multivariable LASSO-regularized Cox models with corresponding coefficients contributing to the radiomic score (RS) for disease-free survival (DFS).

| Radiomic Features | Coefficient |
| --- | --- |
| Log-sigma-0-5-mm-3D_glcm4_Correlation | 2.1645 |
| Log-sigma-0-5-mm-3D_glcm4_MCC | 1.1457 |
| Log-sigma-1-0-mm-3D_glcm4_MCC | 0.8811 |
| Log-sigma-5-0-mm-3D_firstorder_Skewness | 0.3221 |
| Log-sigma-0-5-mm-3D_glszm_smallareahighgraylevelemphasis | 0.0092 |
| Log-sigma-0-5-mm-3D_ngtdm1_Complexity | 0.0056 |
| Log-sigma-1-5-mm-3D_glcm4_clustershade | 0.0005 |
| Log-sigma-0-5-mm-3D_glcm4_clusterprominence | 0.0001 |
| Wavelet-HL_glszm_graylevelvariance | 0.0020 |
| Wavelet-HL_firstorder_Median | -0.0017 |

**Supplementary Figure 1.** Longitudinal dynamics of *EGFR* variant allele frequency (VAF) in circulating free DNA (cfDNA).

VAF levels (Y-axis) are shown for patients with EGFR-activating mutations at baseline (T0) and subsequent follow-up intervals (T1–T4). Individual patient trajectories are color-coded according to the legend.


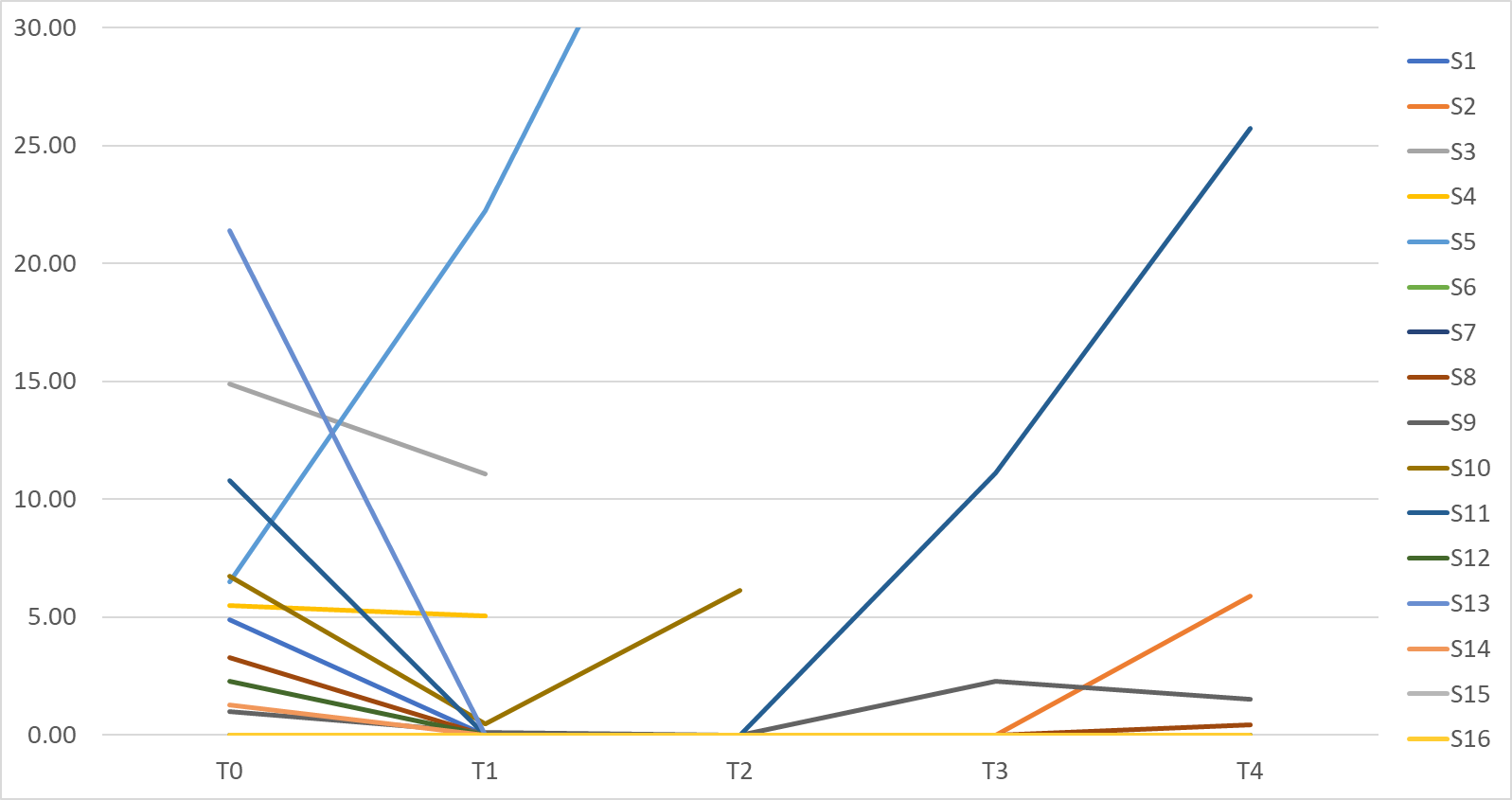

Supplement: Multimedia component 1 [file mmc1.docx]
